# Bispectral index as a predictor of unsalvageable traumatic brain injury

**DOI:** 10.1186/cc14530

**Published:** 2015-03-16

**Authors:** S Mahmood

**Affiliations:** 1 Specialist Anesthesia, Doha, Qatar

## Introduction

The aim was to evaluate the accuracy of bispectral index (BIS) monitoring for the diagnosis of brain death in severely comatose patients. We aimed to determine the utility of the BIS as a tool for clinical evaluation of the moment of brain death.

## Methods

A prospective observational study with waiver of consent was conducted in the trauma ICU for 2 years from October 2012 to September 2014. Monitoring of BIS occurred during patient stay in the ICU. Population: 62 severely comatose patients (Glasgow Coma Score less than 6) admitted to the ICU mainly because of intracerebral hemorrhage, head injury, or postanoxic coma. BIS was recorded continuously during the hospitalization in the ICU. Where necessary, clinical brain death was confirmed by EEG or brain stem test.

## Results

Twenty-nine patients were already clinically brain dead at the time of admission, and their individual BIS values were 0. Twenty-four patients were not clinically brain dead at the time of admission, and their individual BIS values were between 20 and 30. These patients became brain dead, and their individual BIS values dropped to 0 in a few hours to a few days. Seventeen patients who did not become brain dead during their hospitalization in the ICU had persistent electrocerebral activity on EEG, and their average BIS values remained above 31.

## Conclusion

The BIS is a noninvasive, simple, and easy to interpret method, showing a perfect correlation with the other diagnostic methods. BIS can be used in severely comatose patients as an assessment of brain death.

## References

[B1] Paolin A Manuali A Di Paola F Boccaletto F Caputo P Zanata R Reliability in diagnosis of brain death Intensive Care Med 1995 21 657 62 10.1007/BF01711544 8522670

[B2] Gan TJ Glass PS Windsor A Payne F Rosow C Sebel P Bispectral index monitoring allows faster emergence and improved recovery from propofol, alfentanil, and nitrous oxide anesthesia Anesthesiology 1997 87 808 15 9357882 10.1097/00000542-199710000-00014

